# Induction mechanism of ferroptosis: A novel therapeutic target in lung disease

**DOI:** 10.3389/fphar.2022.1093244

**Published:** 2022-12-09

**Authors:** Lingyu Pan, Chunxia Gong, Yehong Sun, Yeke Jiang, Xianchun Duan, Yanquan Han, Yongzhong Wang

**Affiliations:** ^1^ The First Affiliated Hospital of Anhui University of Chinese Medicine, Hefei, Anhui, China; ^2^ College of Pharmacy, Anhui University of Chinese Medicine, Hefei, Anhui, China

**Keywords:** ferroptosis, lung disease, glutathione peroxidase 4, glutathione, reactive oxygen species

## Abstract

Ferroptosis is a newly discovered form of non-apoptotic regulatory cell death driven by iron-dependent lipid peroxidation. Ferroptosis significantly differs from other forms of cell death in terms of biochemistry, genetics, and morphology. Ferroptosis affects many metabolic processes in the body, resulting in disruption of homeostasis, and is related to many types of lung disease. Although current research on ferroptosis remains in the early stage, existing studies have confirmed that ferroptosis is regulated by a variety of genes, mainly involving changes in genes involved in iron homeostasis and lipid peroxidation metabolism. Furthermore, the mechanism of ferroptosis is complex. This review summarizes the confirmed mechanisms that can cause ferroptosis, including activation of glutathione peroxidase 4, synthesis of glutathione, accumulation of reactive oxygen species, and the influence of ferrous ions and p53 proteins. In recent years, the mechanism of ferroptosis in the occurrence and development of many diseases has been studied; the occurrence of ferroptosis will produce an inflammatory storm, and most of the inducing factors and pathological manifestations of lung diseases are also inflammatory reactions. Therefore, we believe that the association between ferroptosis and lung disease deserves further study. This article aims to help readers to better understand the mechanism of ferroptosis, provide new ideas and targets for the treatment of lung diseases, and point out the direction for the development of new targeted drugs for the clinical treatment of lung diseases.

## 1 Introduction

Cell death is an irreversible process that often occurs in normal tissues and is a necessary life process to maintain the function and morphology of tissues. Previous studies have suggested that there are two main pathways of cell death: apoptosis and necrosis. In recent years, a growing number of studies have indicates that there are other types of cell death besides apoptosis and necrosis, such as autophagy, necrotic apoptosis, scorch death, and ferroptosis. In 2003, Dolma et al. (2003) found that cells treated with erastin, a small molecule compound that can be precisely targeted at human cancer cells, did not show the classic characteristics of apoptosis when screening selective lethal anti-tumor drugs in RAS mutated tumor cells. In 2012, Dixon et al. (2012) named this type of death as ferroptosis, an iron-dependent non-apoptotic form of cell death.

Regarding the common cell morphologies in ferroptosis that distinguish it from apoptosis, necrosis, and autophagy, during the process the size of the mitochondria decreases, the density of bilayer membranes increases, and the mitochondrial cristae decrease or disappear; however, the cell membrane remains intact, the size of nucleus is normal, and there is no evidence of nuclear concentration or chromatin marginalization (Peng et al., 2021). In terms of biochemistry, ferroptosis is mainly manifested by the exhaustion of glutathione (GSH), a decline in glutathione peroxidase 4 (GPX4) activity, and the inability of lipid peroxides to be metabolized through the GSH reduction reaction catalyzed by GPX4. Then, ferrous ions (Fe^2+^) mediate lipid oxidation in a manner similar to the Fenton reaction to produce a large number of reactive oxygen species (ROS) (Park and Chung, 2019; Wang et al., 2020). Ferroptosis intersects with multiple cell death pathways were shown by many studies. Furthermore, the working mechanism of ferroptosis is being revealed and clarified underway.

Ferroptosis cells trigger the innate immune system by releasing inflammation related damage factors, and then stimulate the inflammatory response. Pulmonary diseases, such as asthma, acute lung injury, COPD, and pulmonary fibrosis, are closely related to inflammatory response. Therefore, based on the inducing the operating mechanism of ferroptosis, the exploration on the relevance between ferroptosis and lung diseases will conribute to a deeper perspective on pathogenesis of asthma, COPD and other lung diseases, and provide new ideas and directions in treatment methods and new drug research and development.

This review aims to improve our comprehension on the ferroptosis working mechanism so as to provide novel insights during the process of the clinical treatment of lung diseases, and identify the direction for the development of new targeted drugs for the clinical treatment of lung diseases.

## 2 The mechanism of the induction of ferroptosis

### 2.1 Suppression of GPX4

**FIGURE 1:**
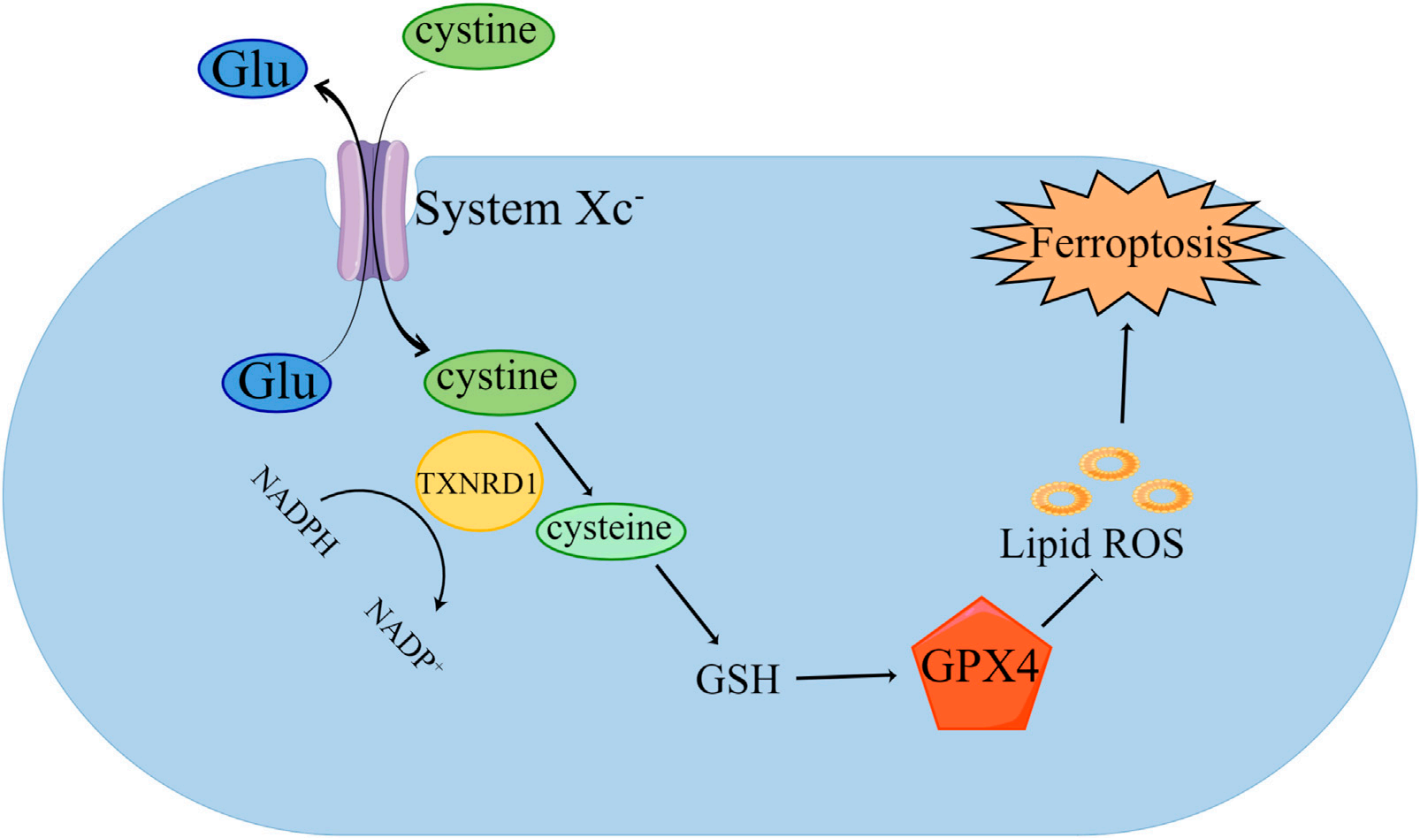
Inhibition of GPX4 induced ferroptosis. Cystine is transported into cells by System xc—and transformed into cysteine by TXNRD1, which further synthesizes GSH. GPX4 uses GSH to remove the phospholipid peroxide (PLOOH). The decrease of GSH leads to the weakening of GPX4’s scavenging effect on lipid peroxides, thus inducing ferroptosis.

GPX4 plays an essential part in ferroptosis in ferroptosis (Figure 1) (Zhang et al., 2021b). Studies have shown that inhibiting the expression of GPX4 can induce iron- dependent cell death (Ding et al., 2021). GPX4 is required to participate in ferroptosis through the induction of erastin and RSL3 (Sui et al., 2018; Zhang et al., 2020b). GPX4 is a GSH-dependent enzyme, which can convert reduced GSH into oxidized GSH, reduce lipid hydroperoxide into lipid alcohol, and convert free hydrogen peroxide into water to reduce iron-dependent lipid peroxidation (Liu et al., 2022a; Dang et al., 2022). GPX4 has selenocysteine (Sec) in its structure (Schwärzler et al., 2022). Sec can bind with RSL3, inhibiting the viability of GPX4 protease, resulting in the lipid peroxides accumulation in cells, which mediates ferroptosis (Shin et al., 2018; Wang et al., 2021). The activity of GPX4 is affected by Sec. As an essential donor in the mevalonate pathway (MVA), the Sec tRNA is modified by isopentenyltransferase. Therefore, inhibition of the MVA can lead to Sel RNA maturation disorder, affecting the normal function of GPX4, and inducing ferroptosis (Chen et al., 2021).

### 2.2 Inhibition of GSH synthesis

GSH is an important antioxidant in cells; it plays a protective role as it can reduce excessive lipid peroxide in cells (Lotocki et al., 2021; Sun et al., 2021). When the synthesis of GSH is blocked, the systems of intracellular oxidation and antioxidant are in a state of out-of-balance, and a large amount of polyunsaturated fatty acid peroxidation cannot be cleared in time; therefore, ferroptosis will occur. The synthesis of GSH depends on System Xc^−^, which is anchored on the cell membrane and consists of seven members of the solute carrier family 11 (SLC7A11) and three members of the solute carrier family 2 (SLC3A2) (Dong et al., 2021). It can transfer cysteine into cells to synthesize GSH. Therefore, inhibiting the activity of System Xc^−^ leads to the inhibition of GSH synthesis and the depletion of GSH in cells, which in turn prevents the effective elimination of lipid peroxides in cells and mediates ferroptosis (Ma et al., 2021).

### 2.3 Intracellular Fe^2+^ overload

**FIGURE 2:**
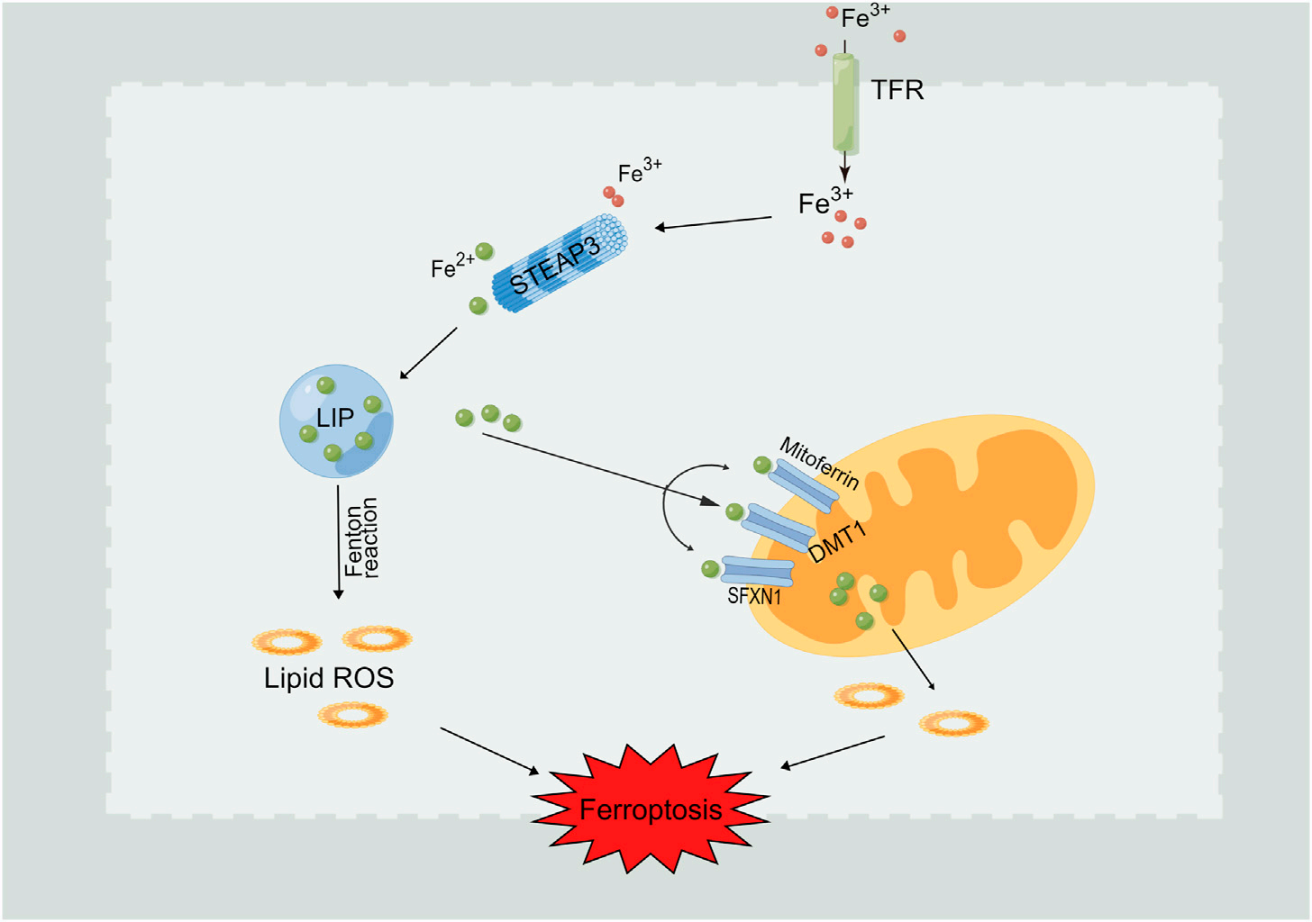
Intracellular Fe^2+^ overload induces ferroptosis. Fe^3+^ flows into the cell through the transferrin receptor (TFR). Fe^3+^is reduced to Fe^2+^ through prostate six times transmembrane protein 3 (STEAP3) and stored in the labile iron pool (LIP). Fe^2+^ can also be further released from the endosome into LIP with the assistance of divalent metal transporter 1 (DMT1). Iron boosts the free radicals generation under the effect of Fenton reaction, which further promoting the occurence of lipid peroxidation and ferroptosis induction.

Normally, the iron ions inside and outside the cell are in a state of dynamic balance. The main iron ion used in cells is Fe^2+^, which is stored in the cell marginal iron pool (LIP) (Krijt et al., 2018). The main routes of iron ions in the LIP are: 1) direct use in cells, 2) enter the mitochondria for biotransformation, 3) bound with ferritin (Fn) and stored in cells, and 4) they are transported out of cells (Xie et al., 2022). As shown in Figure 2, if the cell state is abnormal or the above process is inhibited, it will lead to an overload of Fe^2+^ in the cell. Affected by the Fenton reaction, iron will catalyze the generation of free radicals and promote to the peroxidation of lipid so as to lead to the lipid peroxides accumulation in cells, which eventually brings on the consequence of ferroptosis (He et al., 2020; Fu et al., 2021). In addition, iron reaction element binding protein 2 (IREB2) can significantly increase the expression of ferritin heavy chain and light chain after inhibition, leading to a decreased intracellular free iron level and inhibiting the emergence of ferroptosis (Zhu et al., 2022).

### 2.4 ROS accumulation

The main cause of ferroptosis is the accumulation of lipid peroxides (Gao et al., 2019). Abnormal lipid metabolism leads to the formation of oxidized phospholipids and the accumulation of oxygen free radicals, thus inducing cell death (Zhang et al., 2022). As mentioned above, GPX4 and GSH are inhibited, and the result is the accumulation of ROS, which leads to ferroptosis. Polyunsaturated fatty acids (PUFA) are essential for ferroptosis (Zou et al., 2020), and PUFA is the main substrate for membrane lipid peroxidation. Acyl-CoA synthase long chain family member 4 (ACSL4) and lysophosphatidyltransferase 3 (LPCAT3) play roles in lipid synthesis and modification, respectively. The revelation of ACSL4 and LPCAT3 in ferroptosis resistant cells was significantly decreased (Liao et al., 2022). Furthermore, by downregulating the gene level of ACSL4 and LPCAT3 in cells, the production of lipid peroxides could be effectively prevented, and the ferroptosis resistance could be improved (Reed et al., 2022).

### 2.5 p53 and ferroptosis

p53 is a classical tumor suppressor gene that participates in a variety of cellular metabolic reactions through interactions with other proteins and selective transcriptional regulation of a variety of target genes (Zhao et al., 2021; Zheng et al., 2022). The accumulation of GPX4, GSH, and ROS mentioned above can be regulated by the signaling pathway mediated by p53, thus leading to ferroptosis. p53 can inhibit the activity of solute carrier family 7 member 11 (SLC7A11); cystathionine β synthase (CBS) has a regulatory effect (Hong et al., 2021). p53 can inhibit SLC7A11, reduce the synthesis of GSH through specific pathways, inhibit the viability of GPX4, lead to the accumulation of ROS, and induce ferroptosis (Guan et al., 2020). This discovery has important guiding significance in the treatment of cancer.

## 3 Ferroptosis and lung diseases

### 3.1 Chronic obstructive pulmonary disease (COPD)

COPD is a lung disease characterized by airflow restriction and persistent respiratory symptoms. An abnormal inflammatory reaction caused by exposure to chronic cigarette smoke (CS), harmful particles, or gases is one of the main causes of COPD (Vij et al., 2018). CS is composed of a complex mixture of chemical substances, including free radicals (Zhou et al., 2020). Previous studies have shown that there is iron accumulation in the lungs of long-term smokers, and CS promotes unstable iron accumulation through nuclear receptor coactivator 4-mediated ferritin autophagy, leading to an increase in free iron, and subsequently to phospholipid peroxidation and ferroptosis of pulmonary epithelial cells (Yoshida et al., 2019; Liu et al., 2022b). In a mouse model of CS-induced ferroptosis leading to COPD, GPX4 was shown to be a regulatory target that plays a therapeutic role. Yoshida et al. (2019) reported that accumulation of unstable iron and enhanced lipid peroxidation occurred in cells exposed to CS, and that the accumulation of iron was more severe after GPX4 was knocked out, indicating that ferroptosis may participate in the process of COPD. Furthermore, particle matter 2.5 (PM 2.5) has been shown to induce ferroptosis; the mechanism may be closely related to iron overload, lipid peroxidation, and redox imbalance (Ren et al., 2021).

### 3.2 Acute lung injury

Acute lung injury is characterized by acute systemic inflammation. Its clinical manifestations include pulmonary edema, hypoxemia, diffuse alveolar injury, and lung infiltration (Ren et al., 2021). Acute lung injury can further cause multiple organ dysfunction syndrome or acute respiratory distress syndrome. Acute lung injury is generally caused by extrapulmonary factors and pulmonary inflammation, and its pathogenesis is very complex. Zhou et al. (2019) reported an increase in iron accumulation in the lungs of mice with acute lung injury. The production of superoxide were promoted by the excess iron and leads to lipid peroxidation through the generation of free radicals from the Fenton reaction. The down-regulation of GSH and GPX4 also induces ferroptosis. Liu et al. (2020) showed evidence that ferroptosis plays an important role in lipopolysaccharide (LPS)-induced acute lung injury, and that treatment with the ferroptosis inhibitor ferrostatin-1 (Fer-1) resulted in a significantly improved effect in LPS-induced acute lung injury. Li et al. (2020) listed decreased expression of GPX4 and increased levels of ROS in the cells of mice with acute lung injury induced by radiation; after treatment with liproxstatin-1, an ferroptosis inhibitor, assumed the trend of escalation significantly while the expression of ROS reflected the trend the other way around.

### 3.3 Lung cancer

An increasing number of studies have reported the role of ferroptosis in the pathogenesis and treatment of cancer, including many malignant tumors such as breast cancer (Zhang et al., 2021a), liver cancer (Yang et al., 2020), stomach cancer (Zhang et al., 2020a), rectal cancer (Xian et al., 2020), glioma (Zhuo et al., 2020), and pancreatic cancer (Ye et al., 2021). Typically, immune balance occurs when a tumor is present; the interaction between ferroptosis and lipid metabolism is very important in tumor immune regulation. Researches have proved that the content of prostaglandin-endoperoxide synthase 2 in tumor cells undergoing ferroptosis is abnormally increased, resulting in the production of the immunosuppressive factor prostaglandin E2, thereby inhibiting the tumor immune function of traditional type 1 dendritic cells, natural killer cells, and cytotoxic T cells (Zhao et al., 2022). In recent years, the relationship between lung cancer and ferroptosis has increasingly studied with a focus on NSCLC and lung adenocarcinoma (Tang et al., 2021; Yao et al., 2021).

There is a close relationship between ferroptosis and COPD and lung cancer. COPD caused by excessive smoking is one of the main causes of lung cancer (V et al., 2021). Many substances in CS can induce ferroptosis of airway epithelial cells, which in turn promotes the onset of COPD in lung cancer patients. Research has shown that the combined use of erastin and acetaminophen can promote cell apoptosis and ferroptosis and induce NSCLC cell death (Gai et al., 2020). Furthermore, it can decrease the GSH content and increase lipid peroxide levels to abnormal levels in NSCLC cells. In the radiation resistant subtype NSCLC cells, erastin not only can erastin induce ferroptosis, but also partially reduce cell resistance to radiation (Pan et al., 2019).

### 3.4 Pulmonary fibrosis

Ferroptosis is closely related to the pathogenesis of pulmonary fibrosis. The accumulation of iron ions, ROS, lipid peroxides, and inhibition of GPX4 activity features critically in the pathogenesis of pulmonary fibrosis (V et al., 2021). An imbalance of iron and lipid peroxide metabolism often accompanies pulmonary fibrosis (Hanania et al., 2019). Therefore, inhibiting the accumulation of lipid peroxide and iron in cells is an effective measure to prevent iron-dependent death of alveolar cells and the progression of pulmonary fibrosis. Radiation-induced lung fibrosis (RILF) is a serious and life-threatening complication of radiotherapy treatment for lung cancer; symptoms occur in the first few months after radiotherapy and can last for up to 2 years. The latest research shows that ferroptosis is a novel mechanism of radiation-induced cancer cell death. Inducers of ferroptosis cause biological molecule oxidation (such as lipid oxidation) by enhancing ROS generated by radiation, and drive ferroptosis through phospholipid peroxidation (Ye et al., 2020). Li et al. showed that the level of GPX4 was significantly down-regulated in a RILF mouse model, and that liproxstatin-1 could down-regulate transforming growth factor (TGF) by activating the nuclear factor-erthroid factor 2-related factor 2 (Nrf2) pathway-β1 to alleviate RILF (Li et al., 2019).

## 4 Discussion

Since Dixon and others put forward the concept of ferroptosis in 2012, it has received increasing research attention, with a particular focus on the mechanism. Ferroptosis is a novel form of cell death that disticts from common modes such as apoptosis and autophagy; the occurrence of ferroptosis is regulated by iron metabolism-related mechanisms. This controllable regulatory cell death mode provides new opportunities for the treatment of human diseases.

With increased research, the inducers, inhibitors, and related mechanisms of ferroptosis have been identified. From the perspective of biochemistry, the cause of ferroptosis can be contributed to GSH depletion, intracellular iron accumulation, lipid peroxidation and GPX4 inactivation. In recent years, more and more inducers and inhibitors of ferroptosis have been identified. For example, Nrf2 and heat shock proteins can regulate ferroptosis. Furthermore, the results of a large number of clinical trials and animal experiments indicates that ferroptosis can be closely attributes to a variety of human diseases and pathological processes. The treatment of pathway intervantion proves effectively delay on the process of certain diseases and relieves the symptoms (Chang et al., 2021). Although research on ferroptosis is far from complete at present, the research on ferroptosis and lung diseases has made some progress, Sorafenib, the representative of ferroptosis inducer, has been used as an anti-tumor drug in the treatment of liver and kidney cancer (Sun et al., 2017). RSL3 is one of the representative drugs of GPX4 inhibitors, which can directly target GPX4. RSL3 can trigger ferroptosis by directly inhibiting GPX4 activity and inhibit the growth of glioma cells (Wang et al., 2019). The use of ferroptosis inducers as new adjuvants to traditional treatment schemes in lung cancer has been shown to be effective. The research and development of new inducers of ferroptosis and the employment of a variety form of integrated treatments are promising in the field of lung cancer treatment research. Furthermore, inducing or inhibiting ferroptosis may become a new therapeutic method and drug development target in the treatment of other lung diseases. Relatively speaking, research on ferroptosis and acute lung injury, COPD, and pulmonary fibrosis remains very limited at present; however, there is clear evidence to show the relationship that exists between them. Considering that there is still a large gap to be filled regarding the proposed concept of ferroptosis, with future research, the relationship between ferroptosis and many lung-related diseases will become increasingly clear.

In conclusion, ferroptosis is a newly discovered form of cell death. With continuous in-depth research, new mechanisms and regulatory factors have been identified, and the connection with the processes of many diseases has been confirmed. It has important theoretical and practical value in guiding the development of new therapeutic schemes and targeted drugs for various diseases presenting with ferroptosis as the entry point.

## References

[B1] Chang M. Hou Z. Wang M. Yang C. Wang R. Li F. (2021). Single-atom Pd nanozyme for ferroptosis-boosted mild-temperature photothermal therapy. Angew. Chem. Int. Ed. Engl. 60 (23), 12971–12979. 10.1002/anie.202101924 33772996

[B2] Chen W. Fu J. Chen Y. Li Y. Ning L. Huang D. (2021). Circular RNA circKIF4A facilitates the malignant progression and suppresses ferroptosis by sponging miR-1231 and upregulating GPX4 in papillary thyroid cancer. Aging (Albany NY) 13 (12), 16500–16512. 10.18632/aging.203172 34153004 PMC8266339

[B3] Dang R. Wang M. Li X. Wang H. Liu L. Wu Q. (2022). Edaravone ameliorates depressive and anxiety-like behaviors via Sirt1/Nrf2/HO-1/Gpx4 pathway. J. Neuroinflammation 19 (1), 41. 10.1186/s12974-022-02400-6 35130906 PMC8822843

[B4] Ding Y. Chen X. Liu C. Ge W. Wang Q. Hao X. (2021). Identification of a small molecule as inducer of ferroptosis and apoptosis through ubiquitination of GPX4 in triple negative breast cancer cells. J. Hematol. Oncol. 14 (1), 19. 10.1186/s13045-020-01016-8 33472669 PMC7816340

[B5] Dixon S. J. Lemberg K. M. Lamprecht M. R. Skouta R. Zaitsev E. M. Gleason C. E. (2012). Ferroptosis: An iron-dependent form of nonapoptotic cell death. Cell. 149 (5), 1060–1072. 10.1016/j.cell.2012.03.042 22632970 PMC3367386

[B6] Dolma S. Lessnick S. L. Hahn W. C. Stockwell B. R. (2003). Identification of genotype-selective antitumor agents using synthetic lethal chemical screening in engineered human tumor cells. Cancer Cell. 3 (3), 285–296. 10.1016/s1535-6108(03)00050-3 12676586

[B7] Dong H. Xia Y. Jin S. Xue C. Wang Y. Hu R. (2021). Nrf2 attenuates ferroptosis-mediated IIR-ALI by modulating TERT and SLC7A11. Cell. Death Dis. 12 (11), 1027. 10.1038/s41419-021-04307-1 34716298 PMC8556385

[B8] Fu J. Li T. Yang Y. Jiang L. Wang W. Fu L. (2021). Activatable nanomedicine for overcoming hypoxia-induced resistance to chemotherapy and inhibiting tumor growth by inducing collaborative apoptosis and ferroptosis in solid tumors. Biomaterials 268, 120537. 10.1016/j.biomaterials.2020.120537 33260096

[B9] Gai C. Yu M. Li Z. Wang Y. Ding D. Zheng J. (2020). Acetaminophen sensitizing erastin-induced ferroptosis via modulation of Nrf2/heme oxygenase-1 signaling pathway in non-small-cell lung cancer. J. Cell. Physiol. 235 (4), 3329–3339. 10.1002/jcp.29221 31541463 PMC13482668

[B10] Gao M. Yi J. Zhu J. Minikes A. M. Monian P. Thompson C. B. (2019). Role of mitochondria in ferroptosis. Mol. Cell. 73 (2), 354–363. e353. 10.1016/j.molcel.2018.10.042 30581146 PMC6338496

[B11] Guan Z. Chen J. Li X. Dong N. (2020). Tanshinone IIA induces ferroptosis in gastric cancer cells through p53-mediated SLC7A11 down-regulation. Biosci. Rep. 40 (8), BSR20201807. 10.1042/bsr20201807 32776119 PMC7953492

[B12] Hanania A. N. Mainwaring W. Ghebre Y. T. Hanania N. A. Ludwig M. (2019). Radiation-induced lung injury: Assessment and management. Chest 156 (1), 150–162. 10.1016/j.chest.2019.03.033 30998908 PMC8097634

[B13] He Y. J. Liu X. Y. Xing L. Wan X. Chang X. Jiang H. L. (2020). Fenton reaction-independent ferroptosis therapy via glutathione and iron redox couple sequentially triggered lipid peroxide generator. Biomaterials 241, 119911. 10.1016/j.biomaterials.2020.119911 32143060

[B14] Hong T. Lei G. Chen X. Li H. Zhang X. Wu N. (2021). PARP inhibition promotes ferroptosis via repressing SLC7A11 and synergizes with ferroptosis inducers in BRCA-proficient ovarian cancer. Redox Biol. 42, 101928. 10.1016/j.redox.2021.101928 33722571 PMC8113041

[B15] Krijt M. Jirkovska A. Kabickova T. Melenovsky V. Petrak J. Vyoral D. (2018). Detection and quantitation of iron in ferritin, transferrin and labile iron pool (LIP) in cardiomyocytes using (55)Fe and storage phosphorimaging. Biochim. Biophys. Acta. Gen. Subj. 1862 (12), 2895–2901. 10.1016/j.bbagen.2018.09.005 30279145

[B16] Li X. Duan L. Yuan S. Zhuang X. Qiao T. He J. (2019). Ferroptosis inhibitor alleviates Radiation-induced lung fibrosis (RILF) via down-regulation of TGF-β1. J. Inflamm. 16, 11. 10.1186/s12950-019-0216-0 PMC6542066 31160885

[B17] Li Y. Cao Y. Xiao J. Shang J. Tan Q. Ping F. (2020). Inhibitor of apoptosis-stimulating protein of p53 inhibits ferroptosis and alleviates intestinal ischemia/reperfusion-induced acute lung injury. Cell. Death Differ. 27 (9), 2635–2650. 10.1038/s41418-020-0528-x 32203170 PMC7429834

[B18] Liao P. Wang W. Wang W. Kryczek I. Li X. Bian Y. (2022). CD8(+) T cells and fatty acids orchestrate tumor ferroptosis and immunity via ACSL4. Cancer Cell. 40 (4), 365–378.e6. e366. 10.1016/j.ccell.2022.02.003 35216678 PMC9007863

[B19] Liu H. Forouhar F. Seibt T. Saneto R. Wigby K. Friedman J. (2022a). Characterization of a patient-derived variant of GPX4 for precision therapy. Nat. Chem. Biol. 18 (1), 91–100. 10.1038/s41589-021-00915-2 34931062 PMC8712418

[B20] Liu J. Zhang Z. Yang Y. Di T. Wu Y. Bian T. (2022b). NCOA4-Mediated ferroptosis in bronchial epithelial cells promotes macrophage M2 polarization in COPD emphysema. Int. J. Chron. Obstruct. Pulmon. Dis. 17, 667–681. 10.2147/copd.S354896 35386390 PMC8978690

[B21] Liu P. Feng Y. Li H. Chen X. Wang G. Xu S. (2020). Ferrostatin-1 alleviates lipopolysaccharide-induced acute lung injury via inhibiting ferroptosis. Cell. Mol. Biol. Lett. 25, 10. 10.1186/s11658-020-00205-0 32161620 PMC7045739

[B22] Lotocki V. Yazdani H. Zhang Q. Gran E. R. Nyrko A. Maysinger D. (2021). Miktoarm star polymers with environment-selective ROS/GSH responsive locations: From modular synthesis to tuned drug release through micellar partial corona shedding and/or core disassembly. Macromol. Biosci. 21 (2), e2000305. 10.1002/mabi.202000305 33620748

[B23] Ma L. Zhang X. Yu K. Xu X. Chen T. Shi Y. (2021). Targeting SLC3A2 subunit of system X(C)(-) is essential for m(6)A reader YTHDC2 to be an endogenous ferroptosis inducer in lung adenocarcinoma. Free Radic. Biol. Med. 168, 25–43. 10.1016/j.freeradbiomed.2021.03.023 33785413

[B24] Pan X. Lin Z. Jiang D. Yu Y. Yang D. Zhou H. (2019). Erastin decreases radioresistance of NSCLC cells partially by inducing GPX4-mediated ferroptosis. Oncol. Lett. 17 (3), 3001–3008. 10.3892/ol.2019.9888 30854078 PMC6365906

[B25] Park E. Chung S. W. (2019). ROS-mediated autophagy increases intracellular iron levels and ferroptosis by ferritin and transferrin receptor regulation. Cell. Death Dis. 10 (11), 822. 10.1038/s41419-019-2064-5 31659150 PMC6817894

[B26] Peng J. Hao Y. Rao B. Zhang Z. (2021). A ferroptosis-related lncRNA signature predicts prognosis in ovarian cancer patients. Transl. Cancer Res. 10 (11), 4802–4816. AME Publishing Company. 10.21037/tcr-21-1152 35116333 PMC8797490

[B27] Reed A. Ichu T. A. Milosevich N. Melillo B. Schafroth M. A. Otsuka Y. (2022). LPCAT3 inhibitors remodel the polyunsaturated phospholipid content of human cells and protect from ferroptosis. ACS Chem. Biol. 17 (6), 1607–1618. 10.1021/acschembio.2c00317 35658397

[B28] Ren J. Y. Yin B. W. Li X. Zhu S. Q. Deng J. L. Sun Y. T. (2021). Sesamin attenuates PM(2.5)-induced cardiovascular injury by inhibiting ferroptosis in rats. Food Funct. 12 (24), 12671–12682. 10.1039/d1fo02913d 34825691

[B29] Schwärzler J. Mayr L. Radlinger B. Grabherr F. Philipp M. Texler B. (2022). Adipocyte GPX4 protects against inflammation, hepatic insulin resistance and metabolic dysregulation. Int. J. Obes. 46 (5), 951–959. 10.1038/s41366-022-01064-9 35031697

[B30] Shin D. Kim E. H. Lee J. Roh J. L. (2018). Nrf2 inhibition reverses resistance to GPX4 inhibitor-induced ferroptosis in head and neck cancer. Free Radic. Biol. Med. 129, 454–462. 10.1016/j.freeradbiomed.2018.10.426 30339884

[B31] Subramoniam M. Binson V. A. Mathew L. (2021). Detection of COPD and Lung Cancer with electronic nose using ensemble learning methods. Clin. Chim. Acta 523, 231–238. 10.1016/j.cca.2021.10.005 34627826

[B32] Sui X. Zhang R. Liu S. Duan T. Zhai L. Zhang M. (2018). RSL3 drives ferroptosis through GPX4 inactivation and ROS production in colorectal cancer. Front. Pharmacol. 9, 1371. 10.3389/fphar.2018.01371 30524291 PMC6262051

[B33] Sun J. Wei Q. Zhou Y. Wang J. Liu Q. Xu H. (2017). A systematic analysis of FDA-approved anticancer drugs. BMC Syst. Biol. 11 (5), 87. 10.1186/s12918-017-0464-7 28984210 PMC5629554

[B34] Sun L. Dong H. Zhang W. Wang N. Ni N. Bai X. (2021). Lipid peroxidation, GSH depletion, and SLC7A11 inhibition are common causes of EMT and ferroptosis in A549 cells, but different in specific mechanisms. DNA Cell. Biol. 40 (2), 172–183. 10.1089/dna.2020.5730 33351681

[B35] Tang X. Ding H. Liang M. Chen X. Yan Y. Wan N. (2021). Curcumin induces ferroptosis in non-small-cell lung cancer via activating autophagy. Thorac. Cancer 12 (8), 1219–1230. 10.1111/1759-7714.13904 33656766 PMC8046146

[B60] V A. B. Subramoniam M. Mathew L. (2021). Detection of COPD and Lung Cancer with electronic nose using ensemble learning methods. Clin Chim Acta 523, 231–238. 10.1016/j.cca.2021.10.005 34627826

[B36] Vij N. Chandramani-Shivalingappa P. Van Westphal C. Hole R. Bodas M. (2018). Cigarette smoke-induced autophagy impairment accelerates lung aging, COPD-emphysema exacerbations and pathogenesis. Am. J. Physiol. Cell. Physiol. 314 (1), C73-C87–c87. 10.1152/ajpcell.00110.2016 27413169 PMC5866380

[B37] Wang L. Liu Y. Du T. Yang H. Lei L. Guo M. (2020). ATF3 promotes erastin-induced ferroptosis by suppressing system Xc. Cell. Death Differ. 27 (2), 662–675. 10.1038/s41418-019-0380-z 31273299 PMC7206049

[B38] Wang Q. Bin C. Xue Q. Gao Q. Huang A. Wang K. (2021). GSTZ1 sensitizes hepatocellular carcinoma cells to sorafenib-induced ferroptosis via inhibition of NRF2/GPX4 axis. Cell. Death Dis. 12 (5), 426. 10.1038/s41419-021-03718-4 33931597 PMC8087704

[B39] Wang X. Lu S. He C. Wang C. Ge P. Piao M. (2019). RSL3 induced autophagic death in glioma cells via causing glycolysis dysfunction. Biochem. Biophys. Res. Commun. 518 (3), 590–597. 10.1016/j.bbrc.2019.08.096 31445705

[B40] Xian Z. Y. Hu B. Wang T. Cai J. L. Zeng J. Y. Zou Q. (2020). CircABCB10 silencing inhibits the cell ferroptosis and apoptosis by regulating the miR-326/CCL5 axis in rectal cancer. Neoplasma 67 (5), 1063–1073. 10.4149/neo_2020_191024N1084 32567935

[B41] Xie S. S. Deng Y. Guo S. L. Li J. Q. Zhou Y. C. Liao J. (2022). Endothelial cell ferroptosis mediates monocrotaline-induced pulmonary hypertension in rats by modulating NLRP3 inflammasome activation. Sci. Rep. 12 (1), 3056. 10.1038/s41598-022-06848-7 35197507 PMC8866506

[B42] Yang Y. Lin J. Guo S. Xue X. Wang Y. Qiu S. (2020). RRM2 protects against ferroptosis and is a tumor biomarker for liver cancer. Cancer Cell. Int. 20 (1), 587. 10.1186/s12935-020-01689-8 33372599 PMC7720568

[B43] Yao J. Chen X. Liu X. Li R. Zhou X. Qu Y. (2021). Characterization of a ferroptosis and iron-metabolism related lncRNA signature in lung adenocarcinoma. Cancer Cell. Int. 21 (1), 340. 10.1186/s12935-021-02027-2 34217273 PMC8254945

[B44] Ye L. F. Chaudhary K. R. Zandkarimi F. Harken A. D. Kinslow C. J. Upadhyayula P. S. (2020). Radiation-induced lipid peroxidation triggers ferroptosis and synergizes with ferroptosis inducers. ACS Chem. Biol. 15 (2), 469–484. 10.1021/acschembio.9b00939 31899616 PMC7180072

[B45] Ye Z. Zhuo Q. Hu Q. Xu X. Mengqi L. Zhang Z. (2021). FBW7-NRA41-SCD1 axis synchronously regulates apoptosis and ferroptosis in pancreatic cancer cells. Redox Biol. 38, 101807. 10.1016/j.redox.2020.101807 33271455 PMC7710650

[B46] Yoshida M. Minagawa S. Araya J. Sakamoto T. Hara H. Tsubouchi K. (2019). Involvement of cigarette smoke-induced epithelial cell ferroptosis in COPD pathogenesis. Nat. Commun. 10 (1), 3145. 10.1038/s41467-019-10991-7 31316058 PMC6637122

[B47] Zhang H. Deng T. Liu R. Ning T. Yang H. Liu D. (2020a). CAF secreted miR-522 suppresses ferroptosis and promotes acquired chemo-resistance in gastric cancer. Mol. Cancer 19 (1), 43. 10.1186/s12943-020-01168-8 32106859 PMC7045485

[B48] Zhang H. L. Hu B. X. Li Z. L. Du T. Shan J. L. Ye Z. P. (2022). PKCβII phosphorylates ACSL4 to amplify lipid peroxidation to induce ferroptosis. Nat. Cell. Biol. 24 (1), 88–98. 10.1038/s41556-021-00818-3 35027735

[B49] Zhang K. Ping L. Du T. Liang G. Huang Y. Li Z. (2021a). A ferroptosis-related lncRNAs signature predicts prognosis and immune microenvironment for breast cancer. Front. Mol. Biosci. 8, 678877. 10.3389/fmolb.2021.678877 34164433 PMC8215711

[B50] Zhang Y. Swanda R. V. Nie L. Liu X. Wang C. Lee H. (2021b). mTORC1 couples cyst(e)ine availability with GPX4 protein synthesis and ferroptosis regulation. Nat. Commun. 12 (1), 1589. 10.1038/s41467-021-21841-w 33707434 PMC7952727

[B51] Zhang Z. Guo M. Li Y. Shen M. Kong D. Shao J. (2020b). RNA-binding protein ZFP36/TTP protects against ferroptosis by regulating autophagy signaling pathway in hepatic stellate cells. Autophagy 16 (8), 1482–1505. 10.1080/15548627.2019.1687985 31679460 PMC7469536

[B52] Zhao C. Ghosh B. Chakraborty T. Roy S. (2021). Bavachinin mitigates DMH induced colon cancer in rats by altering p53/Bcl2/BAX signaling associated with apoptosis. Biotech. Histochem. 96 (3), 179–190. 10.1080/10520295.2020.1778087 32664769

[B53] Zhao R. Lv Y. Feng T. Zhang R. Ge L. Pan J. (2022). ATF6α promotes prostate cancer progression by enhancing PLA2G4A-mediated arachidonic acid metabolism and protecting tumor cells against ferroptosis. Prostate 82 (5), 617–629. 10.1002/pros.24308 35089606 PMC9303695

[B54] Zheng Q. Lu W. Yan H. Duan X. Chen Y. Zhang C. (2022). Established pulmonary hypertension in rats was reversed by a combination of a HIF-2α antagonist and a p53 agonist. Br. J. Pharmacol. 179 (5), 1065–1081. 10.1111/bph.15696 34599843

[B55] Zhou H. Li F. Niu J. Y. Zhong W. Y. Tang M. Y. Lin D. (2019). Ferroptosis was involved in the oleic acid-induced acute lung injury in mice. Sheng Li Xue Bao 71 (5), 689–697. 31646322

[B56] Zhou J. S. Li Z. Y. Xu X. C. Zhao Y. Wang Y. Chen H. P. (2020). Cigarette smoke-initiated autoimmunity facilitates sensitisation to elastin-induced COPD-like pathologies in mice. Eur. Respir. J. 56 (3), 2000404. 10.1183/13993003.00404-2020 32366484

[B57] Zhu T. Xiao Z. Yuan H. Tian H. Chen T. Chen Q. (2022). ACO1 and IREB2 downregulation confer poor prognosis and correlate with autophagy-related ferroptosis and immune infiltration in KIRC. Front. Oncol. 12, 929838. 10.3389/fonc.2022.929838 36059676 PMC9428356

[B58] Zhuo S. Chen Z. Yang Y. Zhang J. Tang J. Yang K. (2020). Clinical and biological significances of a ferroptosis-related gene signature in glioma. Front. Oncol. 10, 590861. 10.3389/fonc.2020.590861 33330074 PMC7718027

[B59] Zou Y. Henry W. S. Ricq E. L. Graham E. T. Phadnis V. V. Maretich P. (2020). Plasticity of ether lipids promotes ferroptosis susceptibility and evasion. Nature 585 (7826), 603–608. 10.1038/s41586-020-2732-8 32939090 PMC8051864

